# Interplay of GTPases and Cytoskeleton in Cellular Barrier Defects during Gut Inflammation

**DOI:** 10.3389/fimmu.2017.01240

**Published:** 2017-10-05

**Authors:** Rocío López-Posadas, Michael Stürzl, Imke Atreya, Markus F. Neurath, Nathalie Britzen-Laurent

**Affiliations:** ^1^Universitätsklinikum Erlangen, Erlangen, Germany

**Keywords:** epithelium, endothelium, vascular, barriers, gut, junction proteins, inflammation, inflammatory bowel disease

## Abstract

An essential role of the intestine is to build and maintain a barrier preventing the luminal gut microbiota from invading the host. This involves two coordinated physical and immunological barriers formed by single layers of intestinal epithelial and endothelial cells, which avoid the activation of local immune responses or the systemic dissemination of microbial agents, and preserve tissue homeostasis. Accordingly, alterations of epithelial and endothelial barrier functions have been associated with gut inflammation, for example during inflammatory bowel disease (IBD). The discriminative control of nutriment uptake and sealing toward potentially pathological microorganisms requires a profound regulation of para- and transcellular permeability. On the subcellular level, the cytoskeleton exerts key regulatory functions in the maintenance of cellular barriers. Increased epithelial/endothelial permeability occurs primarily as a result of a reorganization of cytoskeletal–junctional complexes. Pro-inflammatory mediators such as cytokines can induce cytoskeletal rearrangements, causing inflammation-dependent defects in gut barrier function. In this context, small GTPases of the Rho family and large GTPases from the Dynamin superfamily appear as major cellular switches regulating the interaction between intercellular junctions and actomyosin complexes, and in turn cytoskeleton plasticity. Strikingly, some of these proteins, such as RhoA or guanylate-binding protein-1 (GBP-1) have been associated with gut inflammation and IBD. In this review, we will summarize the role of small and large GTPases for cytoskeleton plasticity and epithelial/endothelial barrier in the context of gut inflammation.

## Introduction

**Figure 1:**
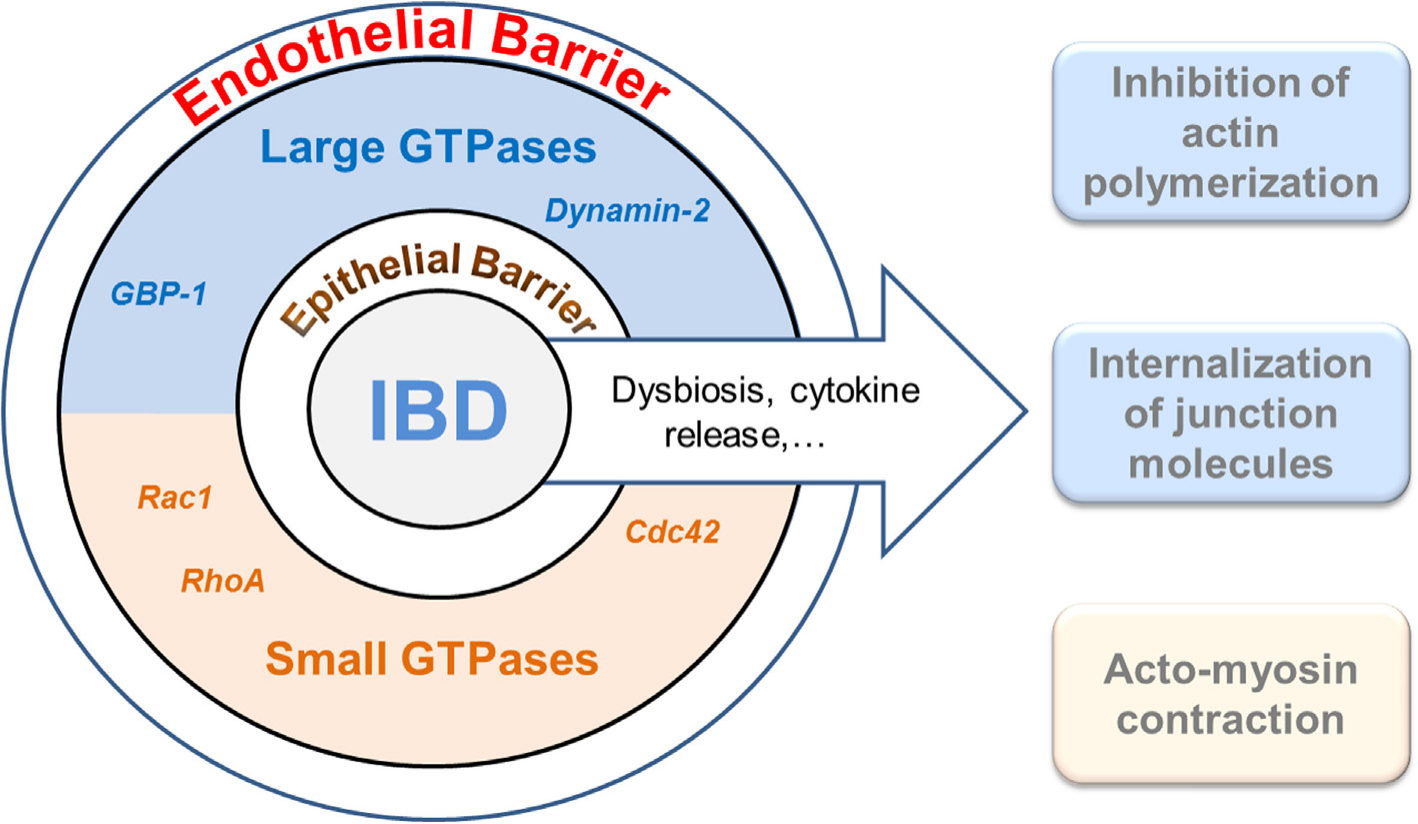
Interplay of GTPases and the cytoskeleton in cellular barrier defects during gut inflammation. The intestinal epithelium and the endothelium establish two coordinated physical and immunological barriers. Increased barrier permeability is pathogenetically associated with inflammatory bowel diseases (IBDs). Different members of the families of small (lower brown) and large (upper blue) GTPases have recently been shown to regulate junctional and cytoskeletal dysfunctions both in epithelial and endothelial cells and, accordingly, may play an important role in IBD. It warrants further studies to determine whether cooperative, antagonistic, or redundant functions are exerted by the different GTPases.

Epithelia at mucosal surfaces represent the first barrier preventing potentially harmful environmental factors to invade the host. In the intestine, the epithelium does not only represent a simple physical obstacle against pathogen invasion but it also regulates nutrient uptake and innate immune function by avoiding the activation of mucosal immune responses (1). Thereby, maintenance of epithelial integrity is a key aspect in order to preserve homeostasis and to impair the development of inflammation in mucosal tissues (2). In addition to the epithelium, the gut–vascular barrier (GVB) has been recently described as a new anatomical structure which builds a second protective barrier preventing the microbiota to enter the bloodstream while allowing the translocation of immune cells and antigens (3). Barrier function of the epithelium as well as of the endothelium is dependent on a complex cytoskeletal organization and, in particular, on the formation of stable cell–cell junctions (4–6). These structures undergo profound changes during inflammation (7). Accordingly, increased paracellular permeability and epithelial/endothelial barrier dysfunction have been linked to the pathogenesis of chronic inflammatory disorders, such as inflammatory bowel diseases (IBDs) (2, 8, 9). IBD is defined as an idiopathic, chronic, and relapsing inflammation of the gastrointestinal tract. Two main clinical manifestations, Crohn’s disease (CD) and ulcerative colitis (UC), affect a rather young population whose quality of life is significantly reduced. Despite intensive research, the pathogenesis of IBD is not completely understood. Here, we discuss the role of small and large GTPases in the cytoskeletal rearrangements induced in intestinal epithelial and endothelial barriers during inflammation (Figure 1).

## Intercellular Junctions in Epithelium and Endothelium

Apical junction complexes (AJC) built by tight junctions (TJs) and adherens junctions (AJs) enable the connection between adjacent cells, both in intestinal epithelium and endothelium. The AJC contribute to barrier function by controlling selective diffusion of molecules or cells, maintaining cell polarity and allowing intercellular communication (10). TJs consist of occludins, claudins, and junctional adhesion molecules (JAMs) (6, 11). AJs are composed of cadherins and nectins (12, 13). Both represent specialized zipper-like structures which enable the sealing of the paracellular space within the epithelial or endothelial layer (14). These intercellular junctions are connected to the actomyosin cytoskeleton *via* cytoplasmatic adaptors, such as zonula occludens (ZO) proteins, and catenins (6, 15, 16), which supports the mechanical strength of the junctions. For instance, in the resting endothelium, the cortical actin network ensures the necessary tension for the formation of stable interactions at AJs (17). AJs and TJs have been shown to influence each other’s assembly and maintenance in a reciprocal manner (18, 19). In the presence of permeability-inducing molecules, actin reorganizes into stress fibers, which increases traction forces and leads to the uncoupling of AJC from the actin cytoskeleton resulting in the formation of gaps between adjacent cells (20, 21). Contraction of a perijunctional actomyosin ring further regulates permeability in a myosin light-chain kinase- dependent manner (22). In addition, TJ and AJ molecules can be removed from the cell surface by internalization and/or by proteolytic cleavage resulting in extracellular domain shedding (18). Thus, the interaction between cytoskeleton and intercellular junctions is crucial for maintenance of epithelial/endothelial barrier function (23).

Intercellular junction composition and abundancy are tissue-dependent. Within the intestinal epithelium, TJs proteins can be categorized in three families: claudins (claudin-1, 2, 3, 4, 5, 7, and 15) (24), tight junction-associated Marvel proteins (Occludin, Marvel D3, and tricellulin) (25), and cortical thymocyte marker of the *Xenopus* (CTX) (JAM-A, CAR, and CLMP) (26). The composition and structure of endothelial TJs can vary according to the type of vessel or organ (27). In intestinal endothelial cells (EndoCs), TJs are composed of occludin, JAM-A, ZO-1, and cingulin, while claudin-5 was mostly associated with gut lymphatic EndoCs (3). Epithelial AJs are composed of α- and β-catenin and E-cadherin, while AJs within EndoCs are formed by VE-cadherin and β-catenin (3). The formation of VE-cadherin adhesions at AJs is the primary event regulating EndoC-cell interactions during vasculogenesis, and this depends on intracellular tension generated by the actin cytoskeleton (18).

## Epithelial Barrier Regulation during Intestinal Inflammation

Epithelial integrity in the gut has to be tightly regulated. In order to build up a protective barrier against luminal content, a precise and complex cell turnover warranties the renewal of the epithelium without compromising its tightness. Stem cells at the crypt bottom proliferate and differentiate into several IECs subtypes with specialized biological functions (28). Then, most of the differentiated IECs migrate upwards to the villus tip, where aged cells die and are shed into the lumen (29, 30). During this sophisticated process, the tightness of the epithelial layer is achieved by the intimate connection between epithelial cells, which is primarily mediated by intercellular junctions connected to the actin cytoskeleton (6). Focusing on cell shedding, the maintenance of epithelial integrity is warranted by the redistribution of junctional proteins along lateral membranes in a cytoskeleton and membrane trafficking-dependent molecular mechanism (31, 32).

The complex cytoskeleton network in IECs (4, 23, 31) orchestrates key cellular and molecular events during epithelial morphogenesis and renewal (12, 33). On a cellular level, the cytoskeleton defines cell shape and polarity which are important for nutrient uptake, anchoring of IECs to the basal membrane and communication with the sub-epithelial compartment (34, 35). Cytoskeletal plasticity within IECs is relevant to maintain barrier integrity and tissue homeostasis. Accordingly, breakdown of epithelial integrity has been observed after disruption of intercellular junctions and cytoskeleton rearrangement, e.g., in the context of infection or inflammation (36–38).

Increased epithelial TJ permeability is a hallmark of tissue alterations observed in the gut of IBD patients (39–43). Although a correlation between permeability and disease activity could be shown in CD patients, for instance (44, 45), the triggering event involved in the breakdown of gut homeostasis is still a matter of controversy. Mouse studies demonstrated that deficiency of single TJ proteins is not associated with pathology due to compensatory mechanisms (46, 47), except for claudin-15 (48). By contrast, it is well accepted that inflammation-derived mediators mediate TJ dysfunction and thereby contribute to the breakdown of epithelial integrity in experimental colitis and IBD. These mediators include cytokines, such as IL-6 (49), IL-13 (50, 51), TNF (52), and type II Interferon (IFN-γ) (53–55). Then, increased intestinal permeability in IBD patients might be secondary to the release of cytokines within the gut mucosa (56, 57). These cytokines then affect paracellular permeability *via* myosin light-chain II-mediated contraction of the prejunctional actin ring, as shown for TNF in IBD patients (23). These observations support the assumption that epithelial integrity breakdown is indeed a consequence of inflammation.

However, recent studies in IBD patients demonstrated that flares of the disease are preceded by increased permeability, which argues for a causative role of the epithelium in the development of intestinal inflammation (41, 58–60). Interestingly, even healthy relatives (61–63) and non-inflamed gut areas in CD patients (64) showed an elevated intestinal permeability. Accordingly, new therapy strategies based on epithelial restoration led to promising results in IBD patients. For instance, therapeutically induced decrease of epithelial permeability by vitamin D (65, 66) or probiotics (67–69), IL-22-triggered mucus production (70) or maintenance of epithelial cell integrity by butyrate (71, 72), or anti-TNF antibody treatment resulted in a clinical amelioration of chronic colitis (73, 74). The remaining open question is which mechanism might regulate cytoskeleton remodeling and epithelial permeability.

## Vascular Barrier Regulation during Intestinal Inflammation

The endothelium consists of a continuous monolayer of EndoCs lining the wall of blood and lymphatic vessels (75). It represents a semipermeable barrier between the bloodstream and the interstitium which regulates nutrient transport, tissue fluid homeostasis, immune cell transmigration (75), and restricts the transport of proteins in an organ-dependent manner (18). Similar to the epithelium, cell–cell junctions are crucial for the barrier role of the endothelium. The loss of EndoC-cell junctions causes a flux of proteinaceous fluid from the bloodstream into tissues, resulting in the development of edema. In addition to cell–cell junctions, coverage of the EndoC layer by pericytes is involved in the endothelial barrier function and was found to regulate permeability of the blood–brain barrier (76, 77).

The intestinal vascular endothelium represents a specialized vascular bed (3, 78). In the intestine, the capillaries are located directly underneath the epithelial layer and organized in gut–vascular units composed of EndoCs, pericytes, and enteric glial cells (3). Interestingly, the resting gut blood endothelium displays different levels of permeability depending on its localization. In the lamina propria, the endothelial permeability is increased compared to the submucosa, allowing the translocation of nutrients and antigens into the bloodstream while limiting enteric bacteria penetration (3).

During IBD, the intestine undergoes profound histological changes, including massive leukocyte infiltration, increased blood vessel density, and edema, which are all linked to vascular function (79–81). During inflammation, the vasculature is activated by inflammatory cytokines (ICs), such as TNF, interleukin-1 β (IL-1β), or IFN-γ, which leads to the expression of leukocytes adhesion molecules and fosters immune cell transmigration. In addition, neo-angiogenesis is induced and correlates with disease severity. More precisely, elevated levels of vascular endothelial growth factor (VEGF) can be found in the inflamed mucosa and in the blood during active IBD (80, 82–84) and vessel density is increased in the intestinal mucosa during IBD and in mouse model of colitis (9). However, inflammatory mediators such as ICs exhibit antiangiogenic activity and the concomitant presence of angiogenic and angiostatic molecules may disturb the physiologic regulation of angiogenesis (85–87). This might explain the disorganized intestinal vasculature observed in IBD, which is characterized by reduced vessel coverage, increased vessel leakiness, edema, and stenosis (81). Furthermore, vessel permeability strongly increases in both acute and chronic DSS-colitis mouse models compared to healthy animals (9). Interestingly, both ICs and VEGF have been shown to increase paracellular permeability of EndoC monolayers in culture (53, 88–90). In particular, high levels of IFN-γ and markers of IFN-γ-activated endothelium, such as ICAM1, VCAM1, MAdCAM, CXCL10, or guanylate-binding protein-1 (GBP-1), can be detected in the gut mucosa of mice during DSS-induced intestinal inflammation (9). In this model, neutralization of IFN-γ resulted in an increased vessel density while vessel permeability decreased (9). Hence, the vascular effects of IFN-γ during IBD might contribute to disease severity by limiting angiogenesis and increasing vessel permeability, ultimately leading to the loss of GVB function. At the molecular level, endothelial (and epithelial) cells treated with IFN-γ undergo remodeling of the actin cytoskeleton and cell–cell junctions, the latter associated with a decrease of ZO-1 expression and internalization of TJ and AJ proteins (55). Further studies are necessary to understand the exact mechanisms of barrier function regulation by IFN-γ.

## Role of Large and Small GTPases in the Regulation of Cytoskeleton Remodeling during Intestinal Inflammation

Large and small GTPases are molecular switches transducing signals from the extracellular compartment to the intracellular machinery. By means of a GTP–GDP-mediated activation cycle (91), these proteins are involved in numerous biological processes, with dramatic impact on cell biology. Most functions of GTPases depend on their association with cellular membranes. The localization of the protein in close proximity to cellular membranes requires a specific posttranslational modification named prenylation. Prenylation consists of the binding of an isoprenoid at the C-terminal end of the target protein and impacts on protein physicochemical properties, subcellular localization, and function (92, 93). New findings demonstrated the important role of large and small GTPases as major cytoskeleton interacting partners and in the regulation of actomyosin dynamics and intercellular junctions (94). Changes in the GTPase activity promote actomyosin dysregulation associated with pathological conditions in several organs (95–97).

Proteins belonging to the Ras superfamily are defined as small GTPases because of their low molecular weight. The Ras superfamily of proteins consists of five families (Ras, Rho, Ran, Rab, and Arf) and more than 160 different members (98). They participate in the regulation of cell proliferation, cytoskeletal dynamics/morphology, membrane trafficking, cellular adhesion, vesicular, and nuclear transport (99–101). Besides the well-described superfamily of small GTPases, the dynamin superfamily of large GTPases represents a group of enzymes involved in pathogen resistance, budding of transport vesicles, division of organelles, cytokinesis, and cytoskeletal rearrangements (102). It comprises dynamins, Mx proteins, OPA, mitofusins, atlastins, and guanylate-binding proteins (GBPs). Large GTPases are characterized by the ability to oligomerize and harbor an oligomerization-dependent GTPase activity (102).

In the following, we will summarize the role of small and large GTPases in cytoskeleton remodeling, epithelial and endothelial integrity, and their relevance in maintenance of barrier functions in the gut.

### Small GTPases

Impaired small GTPase function in the intestinal epithelium is associated with junctional and cytoskeletal dysfunctions (103–105). Numerous *in vitro* studies demonstrated Rho-mediated regulation of the cytoskeleton within epithelial cells (106–111); both up- and downregulation of Rho protein function can alter actomyosin contractility and in turn impair barrier function (112, 113). Actomyosin contraction due to phosphorylation of MLC2 by ROCK is involved in epithelial RhoA signaling, which is required for pathological as well as physiological epithelial cell extrusion (32, 114). The link between RhoA and intestinal inflammation was first shown in 2003, when increased RhoA activation in experimental colitis and patients suffering from IBD was identified (115). In a subsequent study, it was found that Rho-GDP dissociation inhibitor alpha expression was upregulated in CD and UC patients (116). We recently showed that IBD seems to be associated with impaired RhoA function (117). Inflamed areas in the gut of IBD patients depicted an accumulation of RhoA in the cytosol of IECs. This altered subcellular localization could presumably be a sign of RhoA dysfunction, since association to the plasma membrane is required for GTPase activation (118, 119). Furthermore, IEC-restricted lack of RhoA in mice resulted in the development of spontaneous inflammation (117). Interestingly, another recent study demonstrates that lack of Arhgap17, a RhoGTPase activating protein, causes increased epithelial permeability, not leading to spontaneous colitis but increasing the severity of DSS-induced colitis in mice (120). Taking together, RhoA can be considered as an important regulator of epithelial cytoskeleton and homeostasis in the gut. However, the mechanism and regulation of this process is still controversial. Actomyosin contraction due to phosphorylation of MLC2 by ROCK is involved in epithelial RhoA function, but whether RhoA inhibition, activation or both would modify epithelial integrity and permeability is still unclear.

Rac1 and Cdc42 also appear as attractive targets for the regulation of epithelial barrier function. *In vivo* genetic deletions of Cdc42 or Rac1 within IECs are associated with defects on epithelial cell proliferation and/or differentiation (121–124). Interestingly, genetic deletion of Cdc42 in mice resulted in an intestinal phenotype which resembled human microvillus inclusion disease. In the latter, cytoskeleton remodeling appears as a complementary mechanism to Paneth cell differentiation defects, leading to apical junction disorientation and increased intestinal paracellular permeability (123, 124).

Considering the relevance of regulated small GTPase function for cytoskeleton remodeling within IECs, prenylation has emerged as an attractive candidate target in epithelial restoration. Interestingly, IECs from IBD patients show decreased expression of the prenylation-catalyzing enzyme GGTase-Iβ (117). The link between GGTase-I-mediated prenylation and inflammation was confirmed by the dramatic intestinal distortion observed in mice with GGTase-Iβ-deficient IECs, which was ameliorated upon local induction of Rho activation (117). The destruction of intestinal architecture upon epithelial *Pggt1b*, the gene encoding for GGTase-Iβ (geranylgeranyltransferase1 beta subunit) deletion goes along with cytoskeleton remodeling, cell shedding alterations, and increased intestinal permeability. In conclusion, prenylation may represent a novel relevant pathway for maintenance of gut homeostasis and epithelial integrity. Future studies are needed in order to further elucidate the molecular mechanisms related to Rho GTPases and other targets of prenylation within the intestinal epithelium. In this context, a recent study showed that the commensal microbiota can increase intestinal epithelial permeability through the small GTPase ARF4 (125). The expression of ARF4 led to a decrease in the expression of TJ proteins by a mechanism which still has to be determined (125). These results open new perspectives for the understanding of the role of the microbiome in the regulation of intestinal barrier function and in the onset of colitis.

Similar to their function in the epithelium, small GTPases play an essential role in the regulation of the endothelial barrier function through their impact on actin dynamics (126). RhoA activation and subsequent Rock-mediated actomyosin contractility decreases endothelial barrier function upon permeability-inducing compounds, such as thrombin (127). On the other hand, Rac1 and Cdc42 signals are able to counterbalance an increase of endothelial permeability by stabilizing intercellular junctions, decreasing actin contractility, and in turn facilitating the contact between adjacent EndoCs (128, 129). A complex interplay between opposite effects from RhoA and Cdc42/Rac1 and their functional cooperation defines Rho-mediated regulation of endothelial integrity. This crosstalk between RhoA and Rac1 is of particular importance in the context of chronic inflammation. TNF is well known to induce endothelial actin cytoskeleton reorganization and intercellular gaps through a sequential activation of Cdc42, Rac and RhoA (130). In addition, novel findings demonstrated that endosomoal RhoB also controls Rac1-mediated stabilization of the endothelial barrier (131). Despite these observations, so far, little is known about the role of Rho GTPases and prenylation in EndoCs during intestinal inflammation.

### Large GTPases

Among large GTPases, two molecules (dynamin-2 and GBP-1) are of particular importance in the regulation of barrier function. Dynamins are involved in transcellular and paracellular permeability (132). Both, paracellular and transcellular permeabilities are increased in the intestinal epithelium during IBD (133) and are co-regulated in the microvascular endothelium through a compensatory mechanism, involving Rac, Dynamin-2 and actin (132). In general, transcellular permeability is regulated by vesicular transcytosis, which allows the transfer through a cell of macromolecules, such as albumin, by vesicle-mediated endocytosis and exocytosis (134). During transcytosis, invaginations of the plasma membrane (caveolae) are formed and coated by clathrin and actin. Dynamin finally achieves the scission of the nascent vesicle under GTP hydrolysis (134). In addition, Dynamin-2 regulates paracellular permeability through modulation of TJs and AJs. Dynamin-2 is able to bind several AJ and TJ proteins, to link them with the actin cytoskeleton and to ensure the stability of TJs and AJs in the epithelium and the endothelium (135). Furthermore, Dynamins directly interact with actin, foster actin polymerization, and induce actin bundles formation (136). Dynamin-2 is also involved in the maintenance of the apical constriction and the recycling of E-cadherin (137, 138). Dynamin-2 plays a role in barrier maintenance during TNF-induced epithelial shedding (32) and is also involved in the maintenance of the vascular barrier function under hypoxia, by inducing the activity of eNOS (139). Hence Dynamin-2 represents an important regulator of epithelial and endothelial permeability as well as vascular homeostasis.

Members of the human GBP family are involved in immune response against intracellular pathogens and inflammation (140). GBP-1 is the best characterized protein of the seven-member family (140–143). GBP-1 expression is strongly induced by ICs, notably by IFN-γ and has been detected in the inflamed mucosa during IBD (9, 143, 144). GBP-1 has been found to mediate the inhibitory effects of IFN-γ on cell proliferation, migration, and invasion and to inhibit tumor growth and angiogenesis *in vivo* (85, 86, 145–148). More precisely, GBP-1 can reorganize intracellular actin cytoskeleton in epithelial, endothelial, and T-cells (149, 150). GBP-1 directly interacts with β-actin and inhibits actin stress fiber formation, while co-localizing with cortical actin (149, 151). Actin depolymerization, for instance by latrunculin, has been shown to induce Occludin internalization (152). In addition, GBP-1 was found to localize at TJs both in intestinal crypts of patients with CD and UC and in human IEC lines treated with IFN-γ (144). In this model, the silencing of GBP-1 expression led to increased apoptosis, indicating that it exerts a protective role in epithelium homeostasis (144). However, the role of GBP-1 on cell–cell permeability and junction regulation is still not well understood.

Taken together, large and small GTPases, as well as prenylation, represent novel key players for maintenance of gut homeostasis, regulating epithelial and endothelial integrity under physiological and inflammatory conditions (Figure 1). Despite the here described current knowledge in the field, some still open questions encourage the scientific community in this field to fulfill the description of the molecular mechanism behind these observations. It still remains to be determined to which extend the endothelial barrier participate to IBD pathogenesis and whether angiogenesis or endothelial activation contributes the most to the disease. On the other hand, the description of the role of other Rho GTPases, such as Rac1 or Cdcd42, for epithelial integrity; as well as molecular mechanisms regulating prenylation within IECs, should be further investigated. More detailed studies on inflammation-associated cytoskeleton remodeling within IECs and EndoCs might help in the identification of new target structures for an optimized treatment or early diagnosis of IBD.

## Author Contributions

RL-P, MS, and NB-L wrote the manuscript. IA and MN were critically involved in the design of the work and the discussion of the content. All the authors approved the final manuscript.

## Conflict of Interest Statement

The authors declare that the research was conducted in the absence of any commercial or financial relationships that could be construed as a potential conflict of interest.
